# ‘I Felt Like I was Floating in Space’: Autistic Adults’ Experiences of Low Mood and Depression

**DOI:** 10.1007/s10803-020-04638-6

**Published:** 2020-08-24

**Authors:** Amy Louise Jordan, Magdalena Marczak, Jacqueline Knibbs

**Affiliations:** 1 Faculty of Health and Life Sciences, Coventry University, Coventry, UK; 2 Department of Psychology, University of Warwick, Coventry, UK

**Keywords:** Autism, Mental health, Depression, Low mood, Experiences, Interpretative phenomenological analysis

## Abstract

It is recognised that a high proportion of adults on the autism spectrum experience depressive symptoms. However, limited research has explored autistic peoples’ experiences of low mood and depression. The aim of this study was to explore the lived experiences of low mood and depression for adults on the autism spectrum. The study employed Interpretive Phenomenological Analysis to investigate the experiences of 8 adults (7 males and 1 female), aged between 19 and 51, who had a diagnosis of autism without co-occurring learning disabilities, and experienced low mood or depression. All participants recorded their thoughts and feelings in a mood diary for 1 week and participated in a semi-structured interview. Three superordinate themes emerged from the data: ‘*Autism has made me the person I am*’, ‘*I can’t function in the world*’ and ‘*It’s like trying to do accounts on the futures market’: Making sense of emotions*. Findings highlight a need for specialist mental health provision for adults who are on the autism spectrum. Limitations of this study and implications for future research are discussed.

## Introduction

This study explored how adults with autism^1^ experience low mood and depression. Depression is a broad and heterogenous diagnosis characterised by lowered mood, loss of pleasure in most activities and somatic or intellectual changes that affect an individual’s ability to function in daily life (American Psychiatric Association 2013). Depression has been identified as a major public health concern because of its chronic, reoccurring nature and impact on physical health (World Health Organisation 2018).

Individuals with autism are more likely than the general population to experience co-occurring mental health difficulties (Joshi et al. 2013; Smirnoff et al. 2008; Kanne et al. 2009). Depression is the most common mental health condition experienced by autistic people (Ghaziuddin et al. 2002), and compared to typically developing individuals, autistic people are 4-times more likely to experience depression in their lifetime (Hudson et al. 2019). Evidence indicates that the presence of depression may worsen the degree of ASC (Autism Spectrum Condition) related impairment (Roy et al. 2015), negatively impact on support networks (Gold 1993) and increase risk of suicide (Cassidy et al. 2014). Additionally, individuals with ASC have an increased risk of suicide attempts compared with those without ASC (Chen et al. 2017).

The emotional processing and communication difficulties that are typical for people with autism make it challenging to accurately diagnose depression in this population (Leyfer et al. 2006). Symptoms of depression may also present differently for autistic people, with increases in social withdrawal, aggression and crying being more common (Ghaziuddin et al. 2002). Furthermore, depressive symptoms overlap with behaviours typical to ASC (Mazzone et al. 2012). Prevalence rates have therefore been inconsistently reported to range from 5 to 82% (Shtayermman 2007). Further understanding of depression, when experienced by autistic adults, is needed to improve assessment procedures, interventions and support for this population (Wigham et al. 2017).

Previous research has explored the aetiology of depression in the ASC population. Sterling et al. (2008) examined the relationship between severity of ASC symptoms, level of cognitive ability and the occurrence of depressive symptoms in adults with ASC. It was concluded that individuals with ASC, who have an awareness of their social difficulties, are more likely to experience depression than those without awareness. Additionally, it was reported that individuals who have an interest in social interaction, but do not have the skills to execute successful social relations, are at increased risk of experiencing depression (Ozonoff et al. 2002). It is therefore possible that having the motivation to form social relationships, and an awareness of an impaired ability to do so, contributes to negative affect (Sterling et al. 2008; Vickerstaff et al. 2007).

Hedley and Young (2006) investigated social comparison processes and depression in children and adolescents with autism. The perception of differences between self and others was reported to be related to depressive symptoms, and it was concluded that social comparison is a prominent factor related to depressive symptoms in this group. Sterling et al. (2008) supported this finding and reported that depression is more common in autistic adolescents as they develop an awareness of societal expectations to establish significant relationships, increase independence, and gain employment. Awareness of a developmental gap between themselves and their peers may therefore be part of the experience of depression for this population. However, this research focused on depression in children and adolescents and therefore lacks generalisability to the adult population.

Penney (2013) explored experiences of four adolescents diagnosed with ASC and co-occurring depression, as well as those of their parents. The participants discussed school related issues, prevention of victimisation and mental health issues. Findings indicated that significant teasing from peers, and an awareness of difference, contributed to the experience of low mood. Additionally, depressive symptoms, such as becoming increasingly withdrawn and losing interest in favourite topics, were reportedly dismissed by professionals as being part of ASC. The young people and their parents therefore felt left to navigate mental health issues on their own, with little support from professionals. As this study focussed on the experiences of adolescents further research is required to develop an understanding of the experiences of adults with ASC and depression.

Capps et al. (1993) pointed to a lack or absence of emotion in people with ASC. However, Jones et al. (2001) used a thematic approach to analyse first-hand accounts of five autistic people, who had published their experiences online, and found evidence to the contrary. This study reported that emotional issues were important to this population and depression was communicated as a significant emotion. Within these accounts, depression was related to a sense of not belonging, difficulties understanding why they were different and being ridiculed by others. As analysis was conducted on accounts that were published online, it was not possible for the researchers to further explore the individuals’ experiences of depression.

### Rationale

Research has uncovered a relationship between social difficulties, awareness of these difficulties, social comparisons and depression. However, research in this area has left the subjective lived experiences of adults with ASC poorly understood. This study therefore seeks to explore how autistic adults experience depression and low mood.

## Method

### Ethical Considerations

Ethical approval was obtained from Coventry University Ethics Committee. Written informed consent was obtained from all participants in accordance with the British Psychological Society Code of Ethics and Conduct (2018).

### Methodological Considerations

Given the aims of this study an Interpretative Phenomenological Analysis (IPA) design was employed. IPA is an appropriate method of analysis because it is concerned with understanding lived experience and how participants themselves make sense of their experiences. As IPA acknowledges potential discrepancies between the participants’ words and the researcher’s interpretations, treats participants as experts, and attempts to equalise the balance of power between autistic informants and non-autistic researchers, it can be argued that it is an effective qualitative approach in autism studies (MacLeod et al. 2018; Howard et al. 2019).

#### Validity

Researcher’s reflexivity is fundamental in the validity of qualitative research as their experiences are brought to the research (Patton 2002). To address the challenge of remaining objective and impartial, all members of the research team engaged with a bracketing interview (Tufford and Newman 2010). The lead researcher also kept a reflective journal throughout the research process to record thoughts and feelings that arose in response to the research process and to reflect on their relationship with the participants.

### Participants

A non-probability, purposive sampling method was adopted, specifically seeking to include adults who had experience of both autism and low mood or depression. A total of seven men and one woman with a formal diagnosis of Asperger’s syndrome^2^ took part in the study. Participant ages ranged from 19 to 51 (*M *= 31.75, *SD *= 12.7). Six participants had received a formal diagnosis of depression, whilst two identified as experiencing low mood that affected their functioning. Table 1 provides further demographic information for the participants.

**Table 1:** Participant characteristics

Participant (pseudonym)	Age	Gender	ASC diagnosis	Timing of diagnosis	Mood	Therapies engaged with
Dawn	35	Female	Asperger syndrome	Adulthood	Diagnosis of depression	Cognitive behaviour therapy; cognitive analytic psychotherapy
Jason	21	Male	Asperger syndrome	Childhood	Diagnosis of depression	None
Dennis	39	Male	Asperger syndrome	Adulthood	Diagnosis of depression	None
Howard	19	Male	Asperger syndrome	Adulthood	Low mood that affects functioning	None
David	20	Male	Asperger syndrome	Adulthood	Diagnosis of depression	Cognitive behavioural therapy
Michael	46	Male	Asperger syndrome	Adulthood	Low mood that affects functioning	Cognitive behaviour therapy
Ryan	23	Male	Asperger syndrome	Childhood	Diagnosis of depression	None
Peter	51	Male	Asperger syndrome	Adulthood	Diagnosis of depression	Cognitive behaviour therapy

### Inclusion and Exclusion Criteria

Adults aged 18 to 65 years with a formal diagnosis of autism, who also identified as having current difficulties with low mood or depression, were eligible to participate in this study. Individuals with formal diagnoses of learning disability or bipolar disorder were excluded from participation. Furthermore, individuals were excluded if they were in receipt of intervention from Crisis Resolution and Home Treatment Services (CRHT). Participants were not excluded if they had received care from a CRHT historically.

### Procedure

#### Community Involvement

Three autistic adults were involved in the design stage of the research. The experts by experience commented on the initial research question and all study materials including the interview schedule, participant information sheet and consent form.

#### Recruitment

Participants were invited to participate through online advertisements posted by Warwick University’s Disability Services and Coventry University’s Health and Wellbeing Department. Potential participants were also informed about the research by clinicians at a private assessment service based within the West Midlands. Eleven potential participants contacted the lead researcher to express an interest in taking part. Three potential participants were excluded due to experiencing depression as part of bipolar disorder.

Eligible participants were invited to attend a preparatory meeting where they were provided with opportunity to ask questions before signing consent. In line with previous autism studies, participants also had opportunity to familiarise themselves with the interview schedule (Cridland et al. 2014; Griffith et al. 2012; Huws and Jones 2015; MacLeod et al. 2018; Petalas et al. 2015). Furthermore, during this meeting participants collected their mood dairy and arranged for interview.

#### Data Collection

All participants took part in a semi-structured interview. Interviews were conducted between 6th August 2018 and 30th November 2018. The location of the interview varied to accommodate the needs of each participant and included the participants’ home (*n *= 1), Warwick University premises (*n *= 2) and Coventry University premises (*n *= 5). Interviews lasted between 38 and 85 minutes (*M *= 56).

Diaries are a useful, less intrusive data collection tool that can be used, in addition to interviews, to enable individuals who struggle to express themselves verbally to communicate their experiences (Humphrey and Lewis 2008; Coates 2002). Participants were invited to complete daily entries in a semi-structured diary for 1 week prior to attending for interview. Participants were encouraged to communicate in their diaries through any medium that conveyed their experience of mood, including drawings. A ‘feelings wheel’ visual aid, designed to help identify feelings, was included in the diary and was also present during interview. All participants made daily entries in their diaries and were invited to refer to their entries during interview.

## Data Analysis

All interviews were audio-recorded and transcribed verbatim. Following transcription, the interview transcripts and diary entries were analysed using IPA. In line with the procedure set out by Smith et al. (2009), the primary researcher began by listening to the first audio recording and reading the corresponding transcript multiple times, whilst writing freely in the margins about initial thoughts, emotions, and interpretations of the content. This technique, ‘free coding’, allowed for the recognition of any preconceptions, and their impact on the researcher’s understanding of the data to be minimised (Elliott et al. 1999). Next, the researcher annotated each transcript line by line highlighting objects of concern; things that mattered to the participant. The focus was on experiential content and on identifying what the objects of concern meant to the participant, to allow for the identification of emerging themes. This process was repeated for each individual transcript and diary entry. The emerging themes were then clustered together, given labels, and the relationships between them were considered. At this point themes that had not been supported by evidence were eliminated.

One participant opted to draw in their diary to communicate regarding their experience of low mood. The participant was invited to discuss their drawing during interview, and a copy of the image has been included in the results to supplement the findings.

### Validity and Credibility

To increase validity of the initial coding, a second researcher provided peer validation by coding transcript extracts. Additionally, themes were co-developed by three members of the research team. Relevant transcript excerpts have been included in the results to validate the emergent themes.

## Results

Three superordinate themes emerged from the analysis. Each superordinate theme consists of three subordinate themes which are outlined in Table 2.

**Table 2:** Superordinate and subordinate themes

Superordinate themes	Subordinate themes
*‘Autism has made me the person I am’*	*(a) ‘I can’t say I’m normal’* *(b) ‘Suddenly I had a reason’: Impact of diagnosis* *(c) ‘I’m proud to say I’m on the spectrum’*
*‘I can’t function in the world’*	*(a) ‘I don’t really have a good enough working knowledge of relationships’* *(b) ‘Trying to act normal’* *(c) Finding ‘Common Connection’*
*‘It’s like trying to do accounts on the futures market’: Making sense of emotions*	*(a) Disconnect between mind and body: ‘It’s like someone has cut the cable’* *(b) ‘Do my best to be a better me’* *(c) ‘And then I had the therapy’*

Table 3 summarises which participants contributed to each subordinate theme.

**Table 3:** Summary of participant contributions to subordinate themes

Participant (pseudonym)	Gender	“Autism has made me the person I am”			“I can’t function in the world”			“It’s like trying to do accounts on the futures market”: making sense of emotions		
		“I can’t say I’m normal”	“Suddenly I had a reason”: Impact of diagnosis	“I’m proud to say I am on the spectrum”	“I don’t really have a good enough working knowledge of relationships”	“Trying to act normal”	Finding “common connection”	Disconnect between mind and body: “It’s like someone has cut the cable”	“Do my best to be a better me”	“And then I had the therapy”
Dawn	Female	✓	✓		✓	✓		✓	✓	✓
Jason	Male	✓	✓	✓	✓		✓	✓	✓	✓
Dennis	Male	✓	✓	✓			✓	✓	✓	
Howard	Male	✓	✓		✓	✓		✓		
David	Male	✓	✓	✓	✓	✓		✓	✓	✓
Michael	Male	✓	✓	✓	✓	✓		✓	✓	✓
Ryan	Male	✓	✓	✓	✓		✓	✓	✓	
Peter	Male	✓	✓		✓	✓	✓	✓	✓	✓

### ‘Autism has Made Me the Person I Am’

All participants reflected on how autism had shaped their identity.

#### ‘I Can’t Say I’m Normal’

Five participants reflected that, from a young age, they developed an awareness that they were ‘significantly different’ from their peers. Dennis described himself as ‘weird compared to other people’ and reflected that many of his ‘autistic traits’ such as being ‘blunt’, and fixating on limited interests, were ‘not normal in society’. Several participants also shared that they think differently, with David explaining that he ‘computes things differently’ to other people. Across the narratives, there is a sense that awareness of these differences has a negative impact on both their beliefs about themselves, and their mood.

Being on the - adults on the spectrum … I can’t say we’re normal. I can’t say I’m normal. Erm, it’s hard to sort of fit in. Erm, and I think that also has a bit of an impact on having low mood and depression. Especially if you can’t fit in. (Jason)

For Dawn, being unable to ‘fit in with what the world was doing’ contributed to the belief that she is ‘a failed person’ and ‘a loser’. Similarly, Peter talked of how he would often be engaged with ‘negative self-conversation’, in which he would attack and criticise himself for his perceived social failings, thus maintaining his low mood.

Additionally, several participants intimated that being autistic made up a large part of who they were as an individual. Peter shared that autism was at the ‘root’ of his social and emotional difficulties and was therefore not a positive condition to have. Conversely, Dennis viewed autism as only one part of his identity and communicated that he would likely struggle to regulate his emotions even if he was not on the spectrum.

#### ‘Suddenly I had a Reason’: Impact of Diagnosis

All participants spoke about receiving a diagnosis of autism. Six participants reached adulthood before their ASC was recognised, whereas two participants received a diagnosis in primary school. When reflecting on life pre-diagnosis, those who were diagnosed in adulthood described a desire to understand themselves and find a satisfactory explanation for their difficulties.

I have had so much, you know, experience of depression and anxiety from my teen years really. You know, I’d been always looking for a cause or you know, a reason and err, erm, you know, couldn’t really find anything satisfactory. (Peter)

Commonly, participants described how receiving a diagnosis of autism helped contextualise and make sense of their difficulties, including depression. Peter felt that being depressed and anxious ‘stemmed err, you know, in large part from being autistic in the first place’, whilst Michael explained that a diagnosis of autism contextualised his ‘less favourable traits’ and allowed for the development of a more favourable self-opinion.

I said in the past I was either autistic or I was an arsehole. Erm, you know, and it’s nice that there might be a reason. (Michael)

Six participants spoke about a need to process and come to terms with the diagnosis, which was described as ‘poignant’ by Peter. There was a sense that Peter was mourning for the ‘prime of [his] life’ and felt as though he had been held back from achieving his full potential.

Having that diagnosis at such a late stage, you know, erm, you know when the prime of life is in the past really so err, that was erm, I think I needed help as well. (Peter)

Both Ryan and Jason were diagnosed in childhood and reflected more on the impact of diagnosis on others’ understanding of their difficulties, rather than their own. They perceived others to be less judgemental and more understanding towards their behaviours, particularly within their school environments. On the contrary, Dennis communicated that being labelled as having ‘high functioning autism’ perpetuated others’ misunderstanding of his difficulties, as they assumed that being autistic did not significantly impact his ability to function.

#### ‘I’m Proud to Say I Am on the Spectrum’

Several participants reflected that there are benefits to being autistic. For example, Michael reflected that ‘autism has made [him] a very driven and high functioning individual’, whilst David noted that he had ‘intrinsic technical abilities’ that had served him well. Ryan also attributed his positive personality traits, such as being ‘honest’ and ‘moralistic’, to being autistic.

What it’s like to have autism though is interesting. Because a lot of people see it as a negative, I don’t really see it as a negative. Even though it has negative connotations in social settings I think that there’s more benefits to the condition than there are drawbacks. (Ryan)

Jason echoed Ryan’s sentiment and stated that he is ‘proud to say [he is] on the spectrum’. There is a sense that the unique qualities possessed as a result of being on the spectrum are integral to the participants’ identity.

### ‘I Can’t Function in the World’

All participants reflected on their experience of interpersonal difficulties and the impact of those experiences on their mood.

#### ‘I Don’t Really Have a Good Enough Working Knowledge of Relationships’

Evident within many of the participants’ narratives was the belief that they cannot, or should not, reach out to others for emotional support, or be honest with them about how they are feeling. Having to be self-reliant is described by Howard as ‘one of [his] biggest problems’, as trying to deal with his problems alone often resulted in his problems becoming more difficult to deal with. Howard reflected that a key barrier preventing him from sharing his experiences with friends is his lack knowledge regarding social boundaries.

I didn’t really know what the boundaries are concerning emotional things so I just generally tend not to talk about that sort of stuff. (Howard)

This notion was supported by Michael, who shared that he had felt the need to be ‘fiercely independent’ his whole life, and by Peter, who highlighted that being unfamiliar with the intricacies of relationships leaves him feeling unable to rely on others.

I just don’t really have a good enough working knowledge of relationships really - I can’t count on anybody else. (Peter)

Additionally, Peter highlighted that being ‘isolationist’ was the only way to be ‘fair’ to others, as he held the belief that being relied upon is burdensome for the other. Similarly, Dawn shared that she should ‘tackle things on [her] own’ so as not to be a burden. A need to take personal responsibility and be proactive is therefore evident across participants’ accounts.

Many participants shared that pursuing employment was difficult. Having limited capacity to read social situations contributed to Peter feeling like he was ‘easy meat’ and ‘a target’ in work environments. Dennis also recounted how he had had difficulty maintaining steady employment, and communicated that his employment difficulties were caused by the employers’ failure to make reasonable adjustments. Locating the problem externally, appeared to enable Dennis to continue pursing employment. Conversely, Peter located the problem internally, and experienced high levels of shame regarding his occupational history, resulting in him isolating himself, for fear that others would cast judgement on his lack of success.

I reached a point a long time ago where your employment history becomes a vast negative, erm, you know … so social isolation has increased because I haven’t really been able to manage relations. (Peter)

**Fig. 1:**
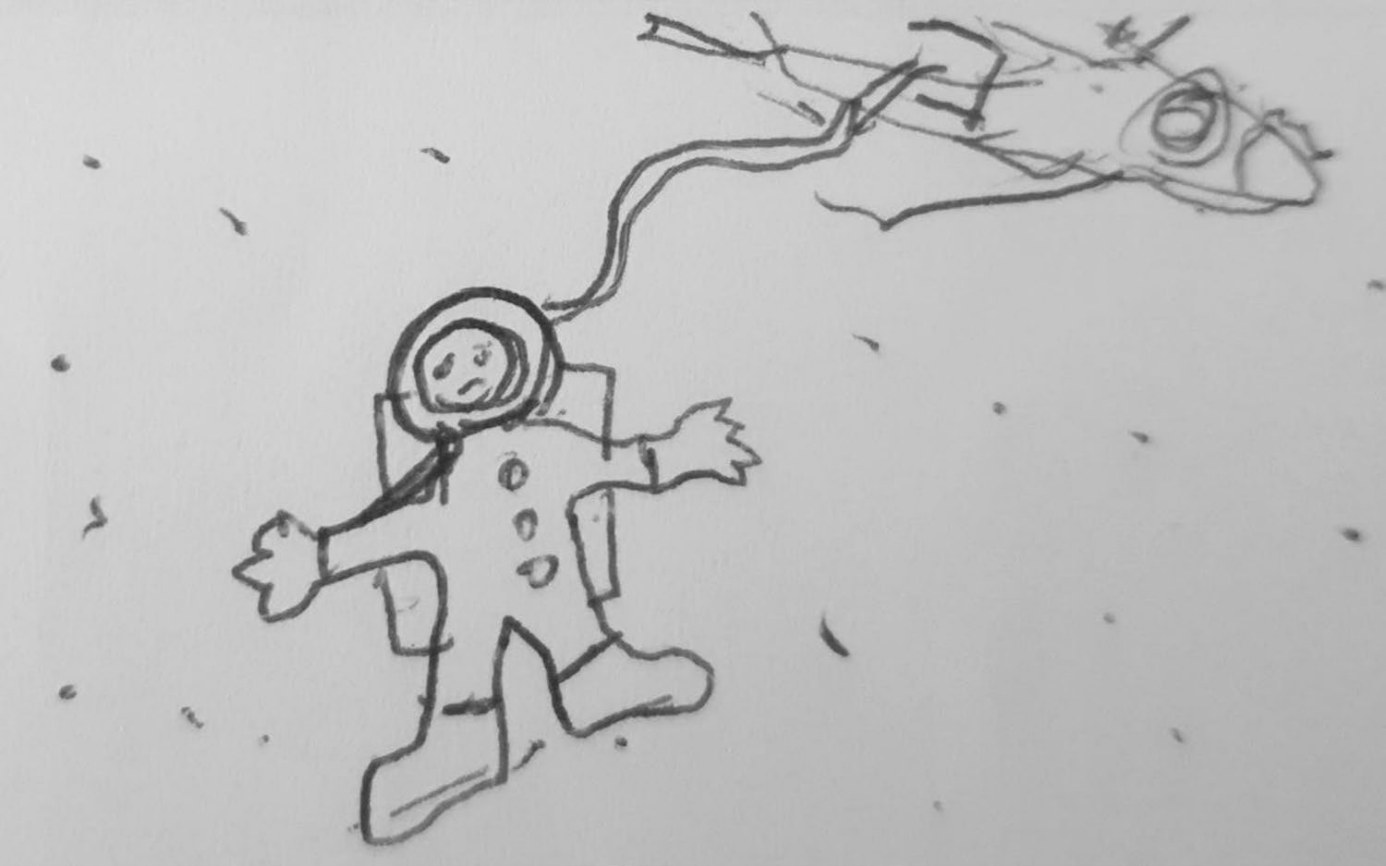
‘I felt like I was floating in space’ as depicted in Dawn’s mood diary

Feeling isolated as a result of interpersonal difficulties was a common theme. Dawn described feeling lonely and isolated even when in the company of others. Dawn’s use of the simile ‘I felt like I was floating in space’ highlighted how disconnected, alone and helpless she can feel in social situations (Fig. 1).

I also felt lonely… isolated because people were talking and having fun but not with me. Erm. And that makes me feel sad because then I thought that people don’t enjoy talking to me so much. Erm, and - and err, that made me feel isolation and fragility. And lonely. [pause] because I felt like I was floating in space. (Dawn)

Furthermore, when discussing her experience of relationships, Dawn highlighted that she lacked the necessary knowledge to enter and sustain a meaningful intimate relationship. There is a sense in her account that being unable to fulfil the desired role of a partner negatively impacted her self-concept.

#### ‘Trying to Act Normal’

Many participants reflected that typically, humans are ‘social animals’, and that to be ‘normal’ they had learned strategies to navigate social interactions. Dawn explains how acting in line with what she ‘should be doing and saying’ serves a social function, but also prevents her from engaging in a way that is authentic, leaving her with a sense of lost identity.

I quite often feel like I’m somebody else that I know or I’ve seen talking or I remember talking with and then I feel like I’m them. Sometimes that’s fine and sometimes it’s not fine. (Dawn)

Several participants also described limiting their interaction with others when feeling low in mood, as their capacity to draw on strategies that they had developed to engage in social interaction was reduced. The perceived need to ‘act normal’ was therefore related to the need to withdraw when feeling low.

When I’m not feeling great I just don’t really have capacity to interact with people normally because I’m thinking about whatever is making me upset, and I just don’t feel like I have any capacity to recall what it is I’ve planned to say in the event of this conversation. (Howard)

Additionally, participants spoke about the need to ‘mask’ or hide their emotions from other people, in order to ‘act normal’. Michael communicated the impact of internalising his emotions in a bid to appear ‘normal’, highlighting how hiding his emotions appeared to perpetuate his low mood.

I think I’ve tried to mask it … I don’t share the fact I’m in a bad mood. But I’m internalising it all. You’re not being honest with yourself or your emotions. I think that is a stressor in itself. (Michael)

#### Finding ‘Common Connection’

Four participants spoke about finding ‘common connection’ with others who identified as autistic or had difficulties with their mental health. Ryan talked about befriending others who have ‘something wrong with them’, as they have shared experiences. Similarly, Jason talked to the importance of being ‘accepted’ and described being able to be his true self when around friends who also have Asperger’s. Dennis also highlighted that his friend who has personal experience of Asperger’s and bipolar is ‘well used to dealing with people that are neuro-atypical’, indicating that having a ‘shared experience’ increases understanding within the relationship, making it easier to sustain.

### ‘It’s Like Trying to Do Accounts on the Futures Market’: Making Sense of Emotions

This superordinate theme arose from discussions of what it is like to feel low in mood or depressed.

#### Disconnect Between Mind and Body: ‘It’s Like Someone has Cut the Cable’

When talking about emotions, several participants described a very cognitive experience, whereby they would attempt to analyse their thoughts and reactions to ascertain what it was they were feeling.

I just think this is what happened I felt x because of y that’s interesting and why don’t I research the neurochemistry behind it. (Howard)

A desire to understand and make sense of their emotional experiences was also evident across the narratives. Michael shared that he felt able to recognise and understand how he was feeling without much difficulty, whereas all other participants spoke about how difficult, or even impossible it was for them to understand emotions.

I guess it’s like doing accounts basically except with emotions. You’ve got to kind of tally up and work out what’s going on except emotions it’s a lot harder to quantify and they’re not always the same and they’re also assumptions. It would be like trying to do accounts on the futures market. (David)

David’s analogy of ‘like trying to do accounts on the futures market’ highlighted that, because emotions are intangible and constantly changing, reconciling them is a difficult, if not impossible task.

Conversely, when talking explicitly about low mood, most participants described a physical embodiment, rather than a cognitive experience.

It’s hard because you don’t exhibit things emotionally, but you do exhibit things physically. I am going to the toilet I am locked up … it’s all sort of locked in … I’m sort of inwardly exhibiting those symptoms and it’s all in the body not in the mind. (Ryan)

Several participants reflected that they felt unable to control their physiological responses, or how their emotions are expressed. This is highlighted by Ryan who shared that when experiencing low mood, it is as though ‘somebody has cut the cable’ between his mind and his body. This difficulty communicating and expressing emotions in a socially appropriate way also increases self-consciousness and fears of being negatively judged by others.

Dennis also highlighted that having difficulty recognising and communicating about emotions is a barrier to being able to seek support.

Asperger’s isn’t a lack of emotion, it’s an inability to process that emotion, or an ability to express that emotion to other people. (Dennis)

#### ‘Do My Best to be a Better Me’

All participants talked about various coping mechanisms that they had employed to cope with low mood and depression. Commonly, participants spoke about self-improvement and ‘constantly striving’ to achieve. Dawn explained how achieving something was an effective way to ‘offset the negativity’ that often accompanied daily life. David also spoke about being in a ‘constant battle’ to ‘do more’ when talking about coping with low mood, highlighting how difficult it is for him to do so. Jason also reflected that pursing personal and professional development opportunities helped him to be less critical and more accepting towards himself.

It sort of settled with me that I can’t change who I am but I can like definitely do my best to be a better me I suppose. (Jason)

Dennis, Jason and Ryan identified coping mechanisms which they had also found to be adaptive. For example, Dennis shared that joining the gym helped improve his mood, as did eating healthily. Whereas Jason noted that listening to his favourite music helped him cope. Several participants also commented on how talking to and spending time with friends and family helped lift their mood; Dennis shared that ‘putting the world to rights with [his] mates’ was beneficial, whilst Jason found turning to his family and friends for support helpful.

I’ve always been able to bring myself back up with the sort of help and support of family and friends. And sort of knowing that I have people around me to support me and are always there for me. (Jason)

Several participants spoke about their interests in the context of coping. It was evident that being able to pursue their interests was integral to the participants’ wellbeing, and Jason made reference to his interests providing him some relief and escapism.

Gaming for me is my coping mechanism. Erm the sort of erm take myself away from reality and erm [pause] sort of put myself in that position instead. (Jason)

Practical, problem solving approaches to emotional difficulties were discussed, with several participants referring to ‘getting on with stuff’ and not letting emotions ‘come out to play’. Ryan also shared how he compartmentalised different areas of his life to maintain productivity and prevent emotional difficulties from hindering his ability to function.

#### ‘And Then I had the Therapy’

Five participants spoke about seeking professional support for their low mood or depression. Michael, Dawn, Peter, Jason and David all reflected on difficulties they had encountered when accessing or engaging with therapeutic support. Within the narratives a common theme of being dismissed or ignored by services arose.

They were like we- yeah because I wasn’t a threat to myself, so they referred me to the university counsellor and then - but I heard nothing back. (Jason)

David and Dawn both reflected on their experiences of Cognitive Behavioural Therapy (CBT). David’s overall experience of CBT was that it was helpful. However, he recognised that he struggled to complete between-session tasks and that being unable to fulfil the therapist’s expectations negatively impacted his mood. Furthermore, Dawn highlighted how having a poor therapeutic relationship with her therapist exacerbated her problems. Additionally, participants who had accessed therapy felt as though their therapist did not have a good enough understanding of autism. In particular, Dawn felt as though her therapist failed to acknowledge her ‘suggestibility’, or that she may have difficulty communicating.

Give the person time to finish explaining. Don’t put words in their mouth. Don’t assume that you know what they’re trying to say. Give them time to fully express. (Dawn)

## Discussion

This study was conducted to explore the lived experiences of autistic adults who experience low mood and depression. The participants’ accounts revealed new insights regarding experiences that negatively impact mood for adults with autism.

Nearly all participants reflected that they were different from their peers, and that awareness of their differences negatively impacted their beliefs about themselves and their mood. This finding supports previous research that concluded that the perception of difference between self and others, and a sense of not belonging, is related to depressive symptoms (Hedley and Young 2006; Jones et al. 2001). Previous research also highlighted how experiencing feelings of difference, without a real understanding of why, causes individuals to internalise the negative attitudes of others and develop low self-esteem (Davies and Neal 1996).

Punshon et al. (2009) reported that accommodating a diagnosis of ASC is a process that may take several months, if not years, and highlighted how important receiving a diagnosis was to provide an explanation of behaviour. The current study supported these findings, as nearly all participants expressed (1) the belief that being diagnosed helped contextualise their difficulties and contributed to improved self-opinions and (2) a period of readjustment was required to come to terms with the news. Moreover, participants reported pride in their unique abilities and personality characteristics, supporting previous research which highlighted that autistic traits can be helpful, depending on context (Russell et al. 2019).

Acting ‘normal’ was a feat that most participants felt was important. These findings highlighted that engaging in a way that is not authentic can contribute to a sense of lost identity. Additionally, attempting to ‘mask’ or hide difficulties appeared to perpetuate low mood. Furthermore, several participants described withdrawing from social interaction when their ability to ‘act normal’ was impacted by low mood, perpetuating their difficulties. This finding further supports Hedley and Young (2006) who concluded that a sense of loneliness, driven by poor social relationships, contributes to levels of depression. However, two participants, who were both diagnosed during childhood, did not narrate the need to ‘act normal’. For them, having a diagnosis of ASC served as an explanation of their differences for others, thus mitigating the need to ‘act normal'. Participants that were diagnosed later in life however described the need to try and fit in, as they did not yet have an explanation for their differences.

All participants reflected on the impact of interpersonal difficulties on their mood. Common amongst participant narratives was the belief that they did not have a good enough understanding of relationships. As a result, many participants chose to be self-reliant and experienced feeling alone and isolated. These findings support the assertion that increased levels of negative affect may be related to poor quality social relationships for people with ASC (Whitehouse et al. 2008). These findings also support Gable and Sheen (2000) who highlighted that lower self-perceived social competence predicts depression.

Several participants made connections between their perceived limited capacity to read social situations, employment difficulties and subsequent negative affect. This finding supports Hurlbutt and Chalmers (2004) who reported that many people with ASC experience difficulties gaining and maintaining employment. Analysis of these accounts indicated that high levels of shame, and consequent withdrawal from social situations, can accompany difficulties maintaining employment. These findings corroborate previous research which asserted that employment difficulties negatively affect the wellbeing of autistic adults (Blustein et al. 2013).

Regarding emotions, participants described a very cognitive experience whereby they attempt to develop insight into how they feel by analysing their thoughts and the context around them. Several participants also identified a desire to understand their emotions, but an inability to do so. Many people with ASC report difficulty identifying and describing their emotions (Bird et al. 2010). Within this research, the inability to recognise and communicate emotions was understood as a barrier to seeking support. Similarly, participants within Camm-Crosbie et al. (2018) study reported that having poor emotional literacy made it difficult for them to recognise and communicate that they needed support.

It was evident that several participants experienced difficulties when accessing therapeutic support. It was felt that there is not enough mental health provision, and that clinical staff do not have a good enough understanding of autism, contributing to negative outcomes and perpetuating negative self-beliefs. These experiences are not unique to the participants included in this study; previous research highlighted that receiving support that is not tailored to individual needs contributes to feelings of disempowerment, isolation and hopelessness for autistic adults (Camm-Crosbie et al. 2018).

However, regarding low mood, nearly all participants described a very physical experience. It was reflected that having an inability to control their own physiological reactions increased feelings of self-consciousness and fears of being judged by others. This finding offers new insights into how low mood is experienced for this population.

Additionally, previous research highlighted that depression in ASC is associated with increased suicidality, with up to 72% of cases reporting suicidal ideation (Zahid and Upthegrove 2017). A history of depression has been identified as a significant risk factor. However, Cassidy et al. (2014) reported that more individuals in their study reported suicidal ideation than had a history of depression, indicating that it is possible that there may be a different route to suicidality for persons with ASC. No themes concerning suicidality were derived from this study, supporting the notion that there may be a different route to suicidality for this population.

### Limitations and Future Research

It is noted that the topic of suicidality was not attended to by participants who participated in this research. As adults with ASC without intellectual disabilities are at highest risk of contemplating suicide (Cassidy et al. 2014), and of dying by suicide (Hirvikoski et al. 2016), it is imperative that future research investigates the route to suicidality for this population.

The findings presented here are based primarily on the experiences of males. It is recognised that the autism phenotype is altered for females, and that females with ASC are typically under-represented in research. Further research is required to explore how autistic females experience low mood and depression. Additionally, three individuals were excluded from this study because they had a diagnosis of bipolar disorder. Although bipolar disorder is being frequently diagnosed amongst adults with ASC (Magan-Maganto et al. 2018) there is a dearth of research on the experience of living with bipolar disorder (Proudfoot et al. 2009). A need for further research in this area is therefore indicated. Future research would also benefit from investigating the role that awareness of one’s own autistic condition has on autistic symptomology, and experience of low mood and depression.

Experts by experience were consulted during the design stage of this study but they were not involved in the data-collection or data-analysis stages of this research. The exclusion of individuals with ASC from meaningful involvement, in all stages of the research process, is problematic and constitutes a barrier to research impact (Milton and Bracher 2013). Further participatory action research, in which people with ASC engage as equal partners throughout the research process, is required (Nicolaidis et al. 2011).

### Clinical Implications

This study indicated that achieving a late-in-life diagnosis can be valuable for adults; it can improve self-awareness and access to limited support. The need for tailored post-diagnostic support, to ensure individuals positively accommodate the diagnosis and identify their individual skills and strengths, is also evident.

Additionally, this research highlighted the need for specialist services that cater to the diverse needs of autistic adults. Clinicians working within mental health services require training to ensure they are knowledgeable about mental health and autism and are able to make reasonable adaptations (Ghaziuddin et al. 2002; Camm-Crosbie et al. 2018).

Furthermore, as many people with ASC have difficulty identifying and describing their emotions, they may benefit from support with emotional literacy prior to engaging with psychological therapies (Camm-Crosbie et al. 2018). Psycho-education regarding emotions, and the link between feelings and physiology, may also reduce the difficulties experienced by individuals who describe embodying their emotions. Moreover, several participants reported that they found the visual aid included with the mood diary incredibly helpful when attempting to identify and describe their emotions. Having access to visual aids may support communication regarding emotions, both inside and outside of therapy.

Finally, participants in this study described difficulties with establishing rapport with their therapist and with completion of in-between session tasks. These findings indicate that more long-term therapy is needed (Anderberg et al. 2017) and that assigned therapeutic tasks should be small. Recruiting a family member to support the individual with therapy may also be beneficial.

## Conclusion

This study has contributed to the limited literature concerning adults’ experiences of ASC and low mood or depression. Supporting findings from previous research, participants shared how awareness of difference, feeling unable to ‘function in the world’, and being unable to communicate a need for and access adequate support contributes to and maintains negative affect. These findings have significant clinical implications for healthcare providers.

## Footnotes

1 There is no one preferred way to talk about autism, and there are different perspectives on the issue within the autism community. To be sensitive to this issue, identity-first language, such as ‘autistic person’, and person-first language, such as ‘person with autism’, is used interchangeably throughout this report.

2 Although Asperger’s syndrome is no longer recognised as a diagnostic label in the Diagnostic and Statistical Manual of Mental Disorders (DSM-5; American Psychiatric Association 2013), all participants were diagnosed with and continued to identify with the label.
